# Association between living in municipalities with high crowding conditions and poverty and mortality from COVID-19 in Mexico

**DOI:** 10.1371/journal.pone.0264137

**Published:** 2022-02-22

**Authors:** Viridiana Ríos, Edgar Denova-Gutiérrez, Simón Barquera

**Affiliations:** 1 Independent Researcher, Mexico City, Mexico; 2 Centro de Investigación en Nutrición y Salud, Instituto Nacional de Salud Pública, Cuernavaca, Morelos, México; Instituto Nacional de Geriatria, MEXICO

## Abstract

**Background:**

The World Health Organization stated a pandemic by severe acute respiratory syndrome coronavirus SARS-Cov2 (COVID-19) on March, 2020 with devastating implications for populations, healthcare systems, and economies globally.

**Objective:**

The present study explores the association between patients living in municipalities with crowding conditions and poverty and mortality from COVID-19 in Mexico; specifically evaluating the socioeconomic characteristics of the municipality in which the patients reside and some individual characteristics.

**Methods:**

In the present study, we examined public information collected from the National Epidemiological Surveillance System informing all persons tested for SARS-CoV-2 and published by the Ministry of Health. The present analysis was restricted to those with the date of registration to October 12, 2021. The association between the main exposures (overcrowded conditions and poverty) and the outcomes of interest (death by COVID-19) was explored using Cox proportional hazard regression models, including frailty penalties to accommodate multilevel data and random effects for the municipality of case occurrence.

**Results:**

A total of 9619917 subjects were included in the Epidemiological Surveillance System for viral respiratory disease platform. Of those for which results were available, 6141403 were negative for COVID-19 and 3478514 were positive for COVID-19; with a total of 273216 deaths in those who tested positive. Among those positive to COVID-19 mean age was 46.9. Patients living in municipalities with high rates of crowding conditions increased the risk of dying from COVID-19 by 8% (95% CI: 1.03, 1.14). Individuals living in municipalities with indigenous background was associated with an increased risk of dying from COVID-19 (HR = 1.10; 95% CI: 1.04, 1.17). Individuals living in municipalities with illiteracy (HR = 1.09; 95% CI: 1.03, 1.11), poverty (HR = 1.17; 95% CI: 1.14, 1.19), food insecurity (HR = 1.094; 95% CI 1.02, 1.06), limited access to social security (HR = 1.10; 95% CI: 1.08, 1.13) and health services (HR = 1.06; 95% CI: 1.04, 1.08) had a higher risk of mortality from COVID-19.

**Conclusion:**

Our data suggest that patients living in municipalities with higher rates of crowding conditions and higher rates of poverty had elevated risk of mortality from COVID-19. In Mexico, the COVID-19 pandemic is a systemic crisis linked to human development since we have seen that it affects less developed and more vulnerable municipalities. Policies to reduce vulnerabilities and develop strategies to deal with health crises like the current one needs to be considered.

## Introduction

The World Health Organization stated a pandemic by severe acute respiratory syndrome coronavirus SARS-Cov2 (COVID-19) in March 2020 [1] with devastating implications for populations, healthcare systems, and economies globally. Understanding the predictors of mortality by COVID-19 is essential for targeting preventing efforts around the world. Extensive literature has proven that age, obesity [2], cardiovascular disease [3, 4], hypertension [5], and type 2 diabetes [6] are established predictors of adverse COVID-19 but we have yet to understand whether socioeconomic determinants play a role on mortality risk.

Based in the “Social Determinants of Health” approach and the ecosocial epidemiology, the cumulative and dynamic interplay processes of exposure, susceptibility, and resistance, which influences health at the singular and general levels, could have been an important factor to understand processes which shape population health—as well as the COVID-19 pandemic [7–11].

Some studies have already suggested that socioeconomic deprivation [12–14], such as crowding conditions [15], living in poorly ventilated spaces [16, 17], and social inequality have a major impact on the transmission of the virus [18]. We also know that these effects may be higher for pregnant women [19]. There is evidence that there is an over-representation of black, Asian and minority ethnic in the COVID-19 positive groups [20–23].

Other communicable and non-communicable disease pandemics show similar socioeconomic patterns [24]. People living in neighborhoods with a high concentration of poverty are more likely to develop diabetes [25, 26], chronic obstructive pulmonary disease [27], and air obstruction [28].

Yet, we do not know what the mechanisms behind such relationship may be. We studied the role of socioeconomic conditions on the effects of COVID-19 by dividing its potential impacts on two mechanisms: those related with living in crowding conditions, and those related directly with income poverty. Closed environments may facilitate secondary transmission of coronavirus disease [17].

The association between COVID-19 and some social determinants in Mexico has been studied [7, 29–32], however, some important questions remain with this topic. Thus, the present study explores the association of living in municipalities with higher rates of crowding conditions and poverty and the risk of mortality from COVID-19 in Mexico. In this sense, we hypothesize that a) the risk of mortality will be higher for individuals living in municipalities with higher rates of crowding conditions and poverty, and b) the risk of mortality will be higher for individuals with obesity, diabetes, and hypertension and living in municipalities with higher rates of crowding conditions.

## Methods

### Study design and data source

In the present study, we examined public information collected from the National Epidemiological Surveillance System (In Spanish, Sistema Nacional de Vigilancia Epidemiológica [SINAVE]) informing all persons tested for COVID-19 and published by the Ministry of Health [33]. This data is updated every day and included: demographic characteristics, certain comorbidities (e.g. Obesity, type 2 diabetes, hypertension, among others), test result (negative or positive to COVID-19), place of residence and whether the patient died. Due to the incidence of severe disease related to COVID-19 and the prevalence of non-communicable diseases is very low in the population under 20 years of age, we only included patients aged 20 years or older [34].

The database contains information of patients who had a COVID-19 test in Mexico at public or private laboratory services and were registered by the Epidemiological Surveillance System for Viral Respiratory Disease (In Spanish, Sistema de Vigilancia Epidemiológica de Enfermedad Respiratoria Viral [SISVER]). Additionally, the patients included could be both hospitalized and ambulatory.

Furthermore, as we reported previously [34], the information included patients from the SISVER in which 475 sentinel surveillance health units (In Spanish, Unidades de Salud Monitoras de Enfermedades Respiratorias [USMER]), across the country test at least 10% of all cases with mild respiratory disease and 100% of severe respiratory disease. Also, patients from hospital base surveillance (no USMER) with severe symptoms of respiratory disease were included (**S1 Fig**).

### Assessment of mortality in patients with a positive diagnosis of COVID-19

For the initial analysis, we only included those who were negative or positive for COVID-19 and who had complete information (S1 Table). In the final analysis, only those who were positive for COVID-19 (laboratory confirmed and diagnosed by using the real-time reverse-transcription polymerase chain reaction method or by rapid Antigen testing) and were recorded as deaths from COVID-19 were included (**S2 Fig**).

### Assessment of overcrowded conditions

To assess social indicators across the study we used the estimations of the Mexican Population Council (In Spanish, Consejo Nacional de Población [CONAPO]) [35] and the Mexican National Council on Evaluation of Social Policy (In Spanish, Consejo Nacional de Evaluación de la Política de Desarrollo Social [CONEVAL]) [36]. Given that at the individual level it is not possible to know directly whether someone lives in crowding conditions, the municipal crowding rate was considered as a probability that the individual lives in high crowding conditions. In this sense, the definition of CONAPO and CONEVAL were followed. For this, two possible measurements were considered at the municipal level. First, the percentage of households that have one bedroom with three or more occupants, or with two bedrooms with five or more occupants. Second, the percentage of households with 2.5 inhabitants or more per room, considering the kitchen but excluding hallways and bathrooms, as measured by the CONEVAL.

### Assessment of municipal level sociodemographic characteristics

These characteristics were divided into three categories: 1) Those related to poverty and income distribution, 2) Those that identify deficiencies and vulnerabilities, and 3) Other specific deficiencies. These characteristics were selected for its possible theoretical relationship with the objective of the study.

#### Poverty and income distribution

Constituted of six different variables: 1) Gini index, which measures the economic inequality of a society, by exploring the level of concentration that exists in the distribution of income among the population. 2) The income below the welfare line, which is equivalent to the total value of the national basic food basket and the non-food basket per person per month. 3) The minimum welfare line, which is equivalent to the value of the food basket per person per month. 4) The percentage of the population of the municipality that lives in a condition of multidisciplinary poverty, defined as the percentage of people that has at least one social deprivation (in the indicators of educational backwardness, access to health services, access to social security, quality and spaces of the house, basic services in the house and access to food) and if their income is insufficient to purchase the goods and services they require to meet their food and non-food needs. 5) The extreme multidisciplinary poverty, defined as the percentage of people who have three or more social deprivations out of six possible and, in addition, their total income is less than the minimum welfare line. The population in this situation has such a low income that even if it were entirely devoted to purchasing food, it would not be able to access those who make up the food basket. And 6) The income ratio which measures the ratio of the average income of the extreme poor population and the average income of the non-poor and non-vulnerable population [35, 36].

#### Deficiencies and vulnerabilities

In this section we included six variables: 1) Percentage of the municipality that has limited access to food, defined as the percentage of people who have moderate to severe food insecurity. 2) Lack of access to social security, defined as the percentage of people who have deficiencies in access to social security according to their age and employment status. 3) Lack of access to basic housing services, defined as the percentage of people who do not meet any of the following four criteria: piped water inside the home or outside the home but on the property; drainage connected to the public water service or to a septic tank; electricity obtained from the public service, from a solar panel or from another source, and that the fuel for cooking is LP gas or natural gas, electricity, and if it is firewood or charcoal that the kitchen has a fireplace. 4) Lack of access to health services, percentage of people who do not have an affiliation or the right to receive health services from any public health institution. 5) Educational backwardness, percentage of people who are of school age and who do not attend school, or if according to their age they have not completed primary or secondary school, according to the criteria indicated above. And 6) Vulnerability due to income, when he/she does not have sufficient income to buy the aggregation of the basic food basket with the basic non-food basket but has no social deprivations.

#### Other specific indicators of vulnerability

Seven additional variables describing specific deficiencies were identified: the percentage of people with illiteracy by municipality, with dirt floors in their homes, who live in localities with less than 5,000 inhabitants, who have incomplete primary school, housing without electricity, housing without a toilet or drainage, and who speak an indigenous language.

### Assessment of individual level characteristics

For this case, we included sociodemographic characteristics (e.g., age, sex, state of birth, state and municipality of residence, nationality, migration status, and, for imported cases, country of origin and native language); we also incorporated event management variables related to COVID-19 like ambulatory or hospitalized management, date of hospitalization (for those who were hospitalized: diagnosis of pneumonia, intubation, and treatment in the intensive care unit), date of symptoms onset, and date of death. Finally, variables related to identification of the case like date of the report, type of testing facility (USMER or not), and healthcare provider were included.

### Assessment of non-communicable diseases

These variables were obtained from SISVER, a public dataset published by the Ministry of Health [33]. Eight variables describe pre-existing non-communicable diseases obtained by self-report like: obesity, hypertension, diabetes, cardiovascular disease, chronic kidney disease (CKD), chronic obstructive pulmonary disease (COPD), asthma, and immunosuppression.

### Statistical analysis

Descriptive analyses of the main characteristics of interest were performed for all patients tested and stratifying by test result (positive or negative to COVID-19). Categorical variables were described as percentages, and continuous variables were defined as means and standard deviations (SDs). In this primary analysis, we compared those who tested positive and negative to COVID-19.

In a second step, we only evaluated patients who tested positive for COVID-19. To analyze this group by tertiles (low, medium and high) of municipalities with information on crowding conditions.

To model COVID-19 mortality risk we computed Cox proportional hazard regression models, including frailty penalties to accommodate multilevel data and random effects for the municipality of case occurrence, approximating iterations using the Newton–Raphson algorithm. Random effect estimates were exponentiated to calculate baseline mortality hazards across municipalities to represent geographical heterogeneity [37]. We tested the joint association of living in municipalities with higher rates of crowding conditions and access to health services with mortality from COVID-19. To assess a possible effect modification, we explored stratified analyses by access to health services and chronic conditions (obesity, type 2 diabetes and hypertension) and living in municipalities with higher rates of crowding conditions.

The statistical analyses were conducted using the Stata Software statistical software package, version 13.0 (StataCorp, College Station, Texas). All P values presented are two-sided; *P* < 0.05 was considered statistically significant.

## Results

### Characteristics of individuals tested for COVID-19

A total of 9619917 individuals who were tested for COVID-19 and were included in the SISVER platform, their characteristics are listed in **S1 Table**. Of those for which results were available, 6141403 were negative for COVID-19 and 3478514 were positive for COVID-19; with a total of 273216 deaths in those who tested positive for COVID-19.

The mean age of study participants was 43.6 years, among those positive to COVID-19, mean age was 46.9 and 40.8 among those negative to COVID-19. Approximately 47.6% of our study population were women. A higher proportion of individuals who tested positive to COVID-19 live in communities with higher indigenous background (7.3 vs 5.0%). Gini index was slightly higher among negative to COVID-19 (41.0 vs 39.0%). Individuals who tested positive to COVID-19 live in communities with higher rates of lack of access to health services (10.7 vs 9.1%) and vulnerability due to income (8.8 vs 8.6%). Prevalence of chronic conditions were higher among those who tested positive to COVID-19.

### Characteristics of individuals tested positive COVID-19

Sociodemographic characteristics of the 3478514 individuals who tested positive for COVID-19 according to levels of crowding conditions are listed in **Table 1**. Compared to those living in municipalities with lower rates of crowding conditions a higher proportion of municipalities with higher rates of indigenous population, population with incomplete elementary school and illiterate was observed among individuals living in municipalities with higher rates of crowding conditions. In terms of poverty and income distribution, Gini index was higher among those who live in municipalities with higher rates of crowding conditions, we also observed that these individuals live in municipalities with higher rates of multidisciplinary poverty and extreme multidisciplinary poverty when compared to the individuals living in municipalities with lower rates of crowding conditions. Gaps and vulnerabilities such as food insecurity, social security and health services were more present in subjects living in municipalities with higher rates of crowding conditions. People living in municipalities with worse crowding conditions were more likely to have a diagnosis of obesity, type 2 diabetes and hypertension compared to those living in municipalities with lower rates of crowding conditions.

**Table 1:** Sociodemographic characteristics and chronic conditions of patients with COVID-19 according to the municipality level of crowding conditions.

	Municipality level of crowding conditions		
Variable	Low n = 1085954	Medium n = 1167697	High n = 1224863
Age	48.3	47.3	49.5
Women, (%)	49.6	51.1	49.5
Indigenous population, (%)	4.8	4.9	5.3
Incomplete Primary school, (%)	18.1	22.3	29.7
Illiteracy, (%)	2.2	4.6	8.0
Gini index, (%)	37.9	39.3	40.5
Lower income welfare line, (%)	33.5	43.1	52.1
Lower income minimum welfare line, (%)	7.2	11.0	17.4
Multidisciplinary poverty, (%)	24.2	33.1	45.2
Extreme multidisciplinary poverty, (%)	1.2	2.6	7.3
Educational backwardness, (%)	9.5	12.3	17.7
Vulnerability due to income, (%)	6.8	9.3	9.9
Limited access to food, (%)	12.2	17.7	24.2
Limited access to social security, (%)	39.6	46.8	59.2
Lack of access to basic housing services, (%)	2.4	5.7	22.9
Limited access to health services, (%)	16.3	17.6	17.7
**Chronic conditions**			
Obesity, (%)	11.9	12.3	13.8
Diabetes, (%)	10.9	11.2	12.5
Hypertension, (%)	14.0	14.7	15.8
Cardiovascular disease, (%)	1.4	1.4	1.6
Chronic obstructive pulmonary disease, (%)	1.1	1.1	1.2
Chronic kidney disease, (%)	1.4	1.4	1.5
Inmunosupresión, (%)	0.8	0.9	1.0
Asthma, (%)	2.4	2.0	2.4

### Role of overcrowding conditions and poverty on mortality

Crude and adjusted hazard ratios and 95% CIs of mortality amongst those who tested positive for COVID-19 are presented in **Table 2**. Living in a municipality with higher rate of crowding conditions increased the risk of dying from COVID-19 by 8% (95% CI: 1.03, 1.14). Living in a municipality with higher rates of indigenous background was associated with an increased risk of dying from COVID-19 (HR = 1.10; 95% CI: 1.04, 1.17). Individuals living in a municipality with higher rates of illiteracy (HR = 1.09; 95% CI: 1.03, 1.11), poverty (HR = 1.17; 95% CI: 1.14, 1.19), food insecurity (HR = 1.04; 95% CI 1.02, 1.06), limited access to social security (HR = 1.10; 95% CI: 1.08, 1.13) and limited health services (HR = 1.06; 95% CI: 1.04, 1.08) had a higher risk of dying from COVID-19.

**Table 2:** Hazard ratios (95% confidence intervals) for mortality amongst those tested positive for COVID-19.

Variables	Crude			Adjusted	
	HR	(95% CI)		HR	(95% CI)
**Municipality rates of crowding conditions**					
Low	1.00	--		1.00	--
Medium	1.01	1.00, 1.02		1.02	1.01, 1.05
High	1.06	1.03 1.08		1.08	1.03, 1.14
P for trend	<0.001			<0.001	
**Municipality rates idigenous population**					
No	1.00	--		1.00	--
Yes	1.16	1.09, 1.24		1.10	1.04, 1.17
P value	<0.001			<0.001	
**Municipality rates of illiteracy**					
Low	1.00	--		1.00	--
Medium	1.01	1.00, 1.02		1.01	0.99, 1.03
High	1.05	1.04, 1.07		1.09	1.03, 1.11
P for trend	<0.001			<0.001	
**Municipality rates of poverty**					
Low	1.00	--		1.00	--
Medium	1.05	1.04, 1.06		1.08	1.07, 1.10
High	1.12	1.11, 1.13		1.17	1.14, 1.19
P for trend	0.001			<0.001	
**Municipality rates of food insecurity**					
Low	1.00	--		1.00	--
Medium	1.01	0.98, 1.03		1.00	0.98, 1.02
High	1.05	1.04, 1.08		1.04	1.02, 1.06
P for trend	<0.001				
**Municipality rates of access to social security**					
High	1.00	--		1.00	--
Medium	1.01	1.00, 1.02		1.02	1.01, 1.04
Low	1.08	1.07, 1.10		1.10	1.08, 1.13
P for trend	<0.001			<0.001	
**Municipality rates of access to health services**					
High access	1.00	--		1.00	--
Middle access	0.99	0.98, 1.00		1.00	0.99, 1.02
Low access	1.04	1.03, 1.05		1.06	1.04, 1.08
P for trend	<0.001			<0.001	
**Age**					
≥ 20 to < 40 years	1.00		--	1.00	--
≥ 40 to < 60 years	5.41		4.30, 7.92	5.13	4.11, 7.35
≥ 60 years	15.90		12.11, 19.90	14.76	11.20, 19.41
P for trend	<0.001				
**Sex**					
Women	1.00		--	1.00	--
Men	1.45		1.30, 1.59	1.40	1.28, 1.54
P value	<0.001			<0.001	
**Municipalities with localities with less than 5,000 inhabitants**					
Yes	1.18		1.10, 1.24	1.14	1.04, 1.25
P value	<0.001			<0.001	

Adjusted for: Age, sex, indigenous population, illiteracy, poverty rate, lack of access to food, lack of social security, lack of health services, Gini index, localities <5 thousand inhabitants, reduced mobility, obesity, diabetes, hypertension, COPD, immunosuppression, asthma, cardiovascular disease, chronic kidney disease.

Adjusted for: Age, sex, indigenous population, illiteracy, poverty rate, lack of access to food, lack of social security, lack of health services, Gini index, localities <5 thousand inhabitants, reduced mobility, smoking, obesity, diabetes, hypertension, COPD, immunosuppression, asthma, cardiovascular disease, chronic kidney disease, time from symptom onset up to death.

### Interaction between living in municipalities with overcrowding conditions, access to health services and chronic conditions

**Fig 1:**
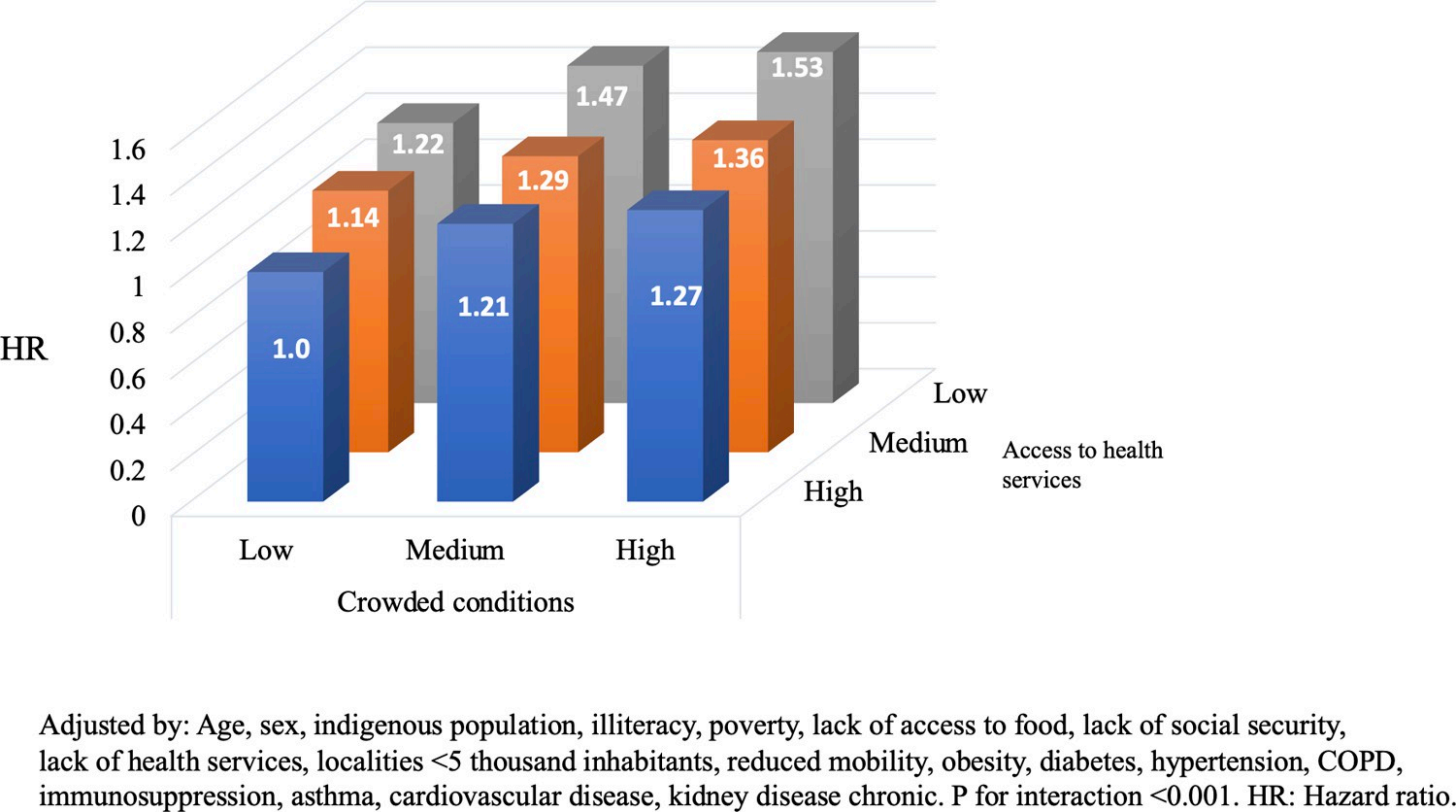
Interaction between crowding conditions and access to health services and mortality on patients with COVID-19.

**Fig 2:**
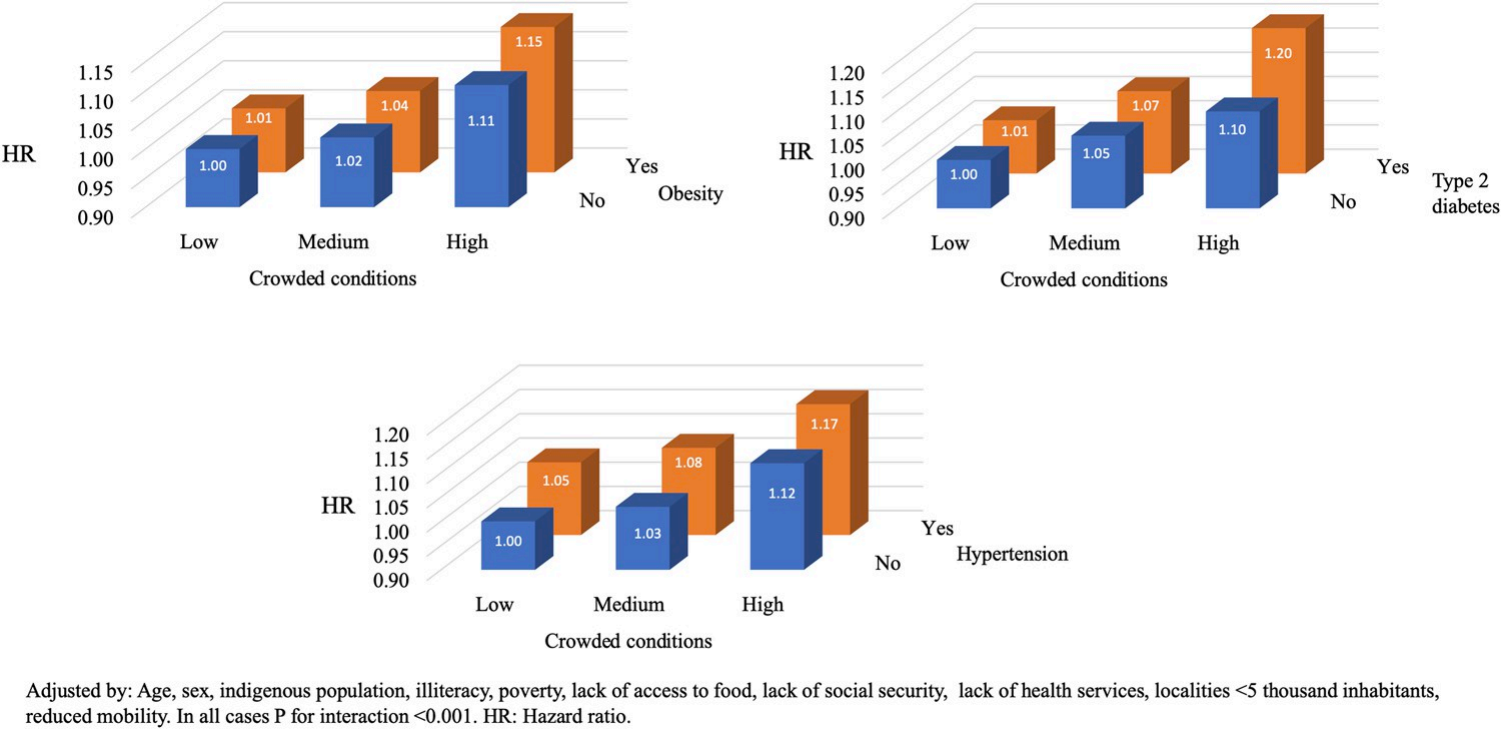
Interaction between crowding conditions and a) Obesity, b) Type 2 diabetes and c) Hypertension and mortality on patients with COVID-19.

We tested the joint association between living in municipalities with higher rates of crowding conditions and access to health services and chronic conditions with mortality from COVID-19 (Figs 1 and 2). In Fig 1, the reference group for comparisons was composed of subjects living in municipalities with lower rates of crowding conditions and subjects living in municipalities with higher rates of access to health services. Relative to the reference group, the HR of the individuals living in a municipality with higher rates of crowding conditions and individuals living in municipalities with lower rates of access to health services was 1.53 (95% CI: 1.40, 1.67; P for interaction <0.001). Additionally, we observed that relative to the reference group, the HR of the group living in a municipality with higher rates of crowding conditions and living with type 2 diabetes was 1.20 (95% CI: 1.14, 1.27; P for interaction <0.001).

## Discussion

The aim of the present study was to explore the association between crowding conditions and poverty and the mortality risk from COVID-19 in Mexico, with additional emphasis in other social determinants like some deficiencies (e.g. food insecurity) and vulnerabilities (e.g. indigenous population, illiteracy), as well as the interaction with some comorbidities. From a total of 3478514 cases who tested positive for COVID-19, our data suggests that municipalities with higher rates of crowding conditions, and higher rates of poverty (measured by the income below the welfare line) are associated with higher risk of mortality from COVID-19 in Mexican adult population. In addition, we also found, that in municipalities with higher rates of conditions like being indigenous, illiterate, food insecure, lack of access to social security, health services, from places with <5,000 inhabitants increase the risk of dying from COVID-19.

Also, there is multiple evidence suggesting that noncommunicable diseases and COVID-19 may be associated at different levels, increasing the probability of severe disease and death [34, 38]. Furthermore, given the high burden of disease attributable to non-communicable diseases in Mexico, it is necessary to generate information on their contribution to the risk of mortality from COVID-19. Our findings suggest that individuals living in a municipality with higher rates of crowding conditions and living with obesity, hypertension, and/or diabetes, were associated to a higher risk of mortality from COVID-19.

Our study suggests that social inequalities, like higher rates of crowding conditions and poverty have a relevant contribution and role in the COVID-19 pandemic in Mexico. Our analysis found that patients living in municipalities with higher rates of crowding conditions had 8% (95% CI: 1.03, 1.14; P trend <0.001) higher risk to die from COVID-19, compared to those living in a municipality with lower rates of crowding conditions. A recent study conducted by Krieger et al. [39] found a similar association; households in the highest crowding group had greater risk ratios (RR = 1.7; 95% CI: 1.0, 2.9) of mortality from COVID19 compared to those in lowest crowding group. In this sense, previous studies have suggested that higher rates of crowding conditions has been related to certain factors such as poor ventilation, air pollution and poor sanitation within homes which has been related with infectious diseases, particularly respiratory diseases [40]. Additionally, other study has proposed that higher rates of crowding conditions could increase anxiety, stress and depressive symptoms which has been related with obesity and other non-communicable diseases via low grade inflammation and this could also explain the relation with COVID-19 [41].

Initially in Mexico, the spread of COVID-19 began in subjects of high socioeconomic status who live in more developed municipalities of the country; however, our data suggest that more vulnerable populations living in small areas, which are exposed to a persistent and historical context of social deprivation, have been more affected by COVID-19. Thus, we observed that patients living in municipalities with higher rates of poverty had 17% (95% CI: 1.14, 1.19; P trend <0.001) higher risk of mortality from COVID-19, compared to those living in municipalities with lower rates of poverty. Consistent with our findings, previous studies have suggested that poverty and other vulnerabilities are associated with an increased risk of mortality from COVID-19 [39, 42–44].

These findings are of concern specially since these populations lack the resources to implement or adopt preventive measures. For example, given their socio-economic conditions, having no financial reserves, and depending on emergency government assistance, they will scarcely be able to adhere to special recommendations such as social isolation, wearing masks, and hand hygiene. Also, the lack of access to minimum resources, such as potable clean water and basic sanitation (e.g. drainage) can increase the risk of illness due to COVID-19, as observed with other respiratory diseases [45].

To our knowledge, there are no previous studies evaluating the joint effect of crowding conditions and lack of access to health services with the risk of mortality from COVID-19. Our results suggest that patients living in municipalities with higher rates of crowding conditions and with lower rates of access to health services had 53% (95% CI: 1.34, 1.72; P for interaction <0.001) higher risk to die from COVID-19, compared with those patients living in municipalities with lower rates of crowding conditions and living in municipalities with higher rates of access to health services.

We also found a joint effect of living in municipalities with higher rates crowding conditions and living with certain comorbidities like obesity, diabetes, and/or hypertension, and the risk of mortality from COVID-19. For example: our data proposed that compared with patients living in municipalities with lower rates of crowding conditions and without obesity, patients living with obesity and living in municipalities with higher rates crowding conditions had 1.15 times higher risk of dying from COVID-19.

As these communities face crowding conditions, poverty, lack of access to health care, food insecurity, lack of access to basic sanitation services, among other deficiencies, there is a need to focus more on the underlying factors that put them at higher risk. For that reason, in these vulnerable groups the governments should place more emphasis on their social distancing behaviors and assess their needs for basic resources such as food, medicine, personal protective equipment and other essential supplies necessary to socially distance themselves during the current pandemic.

COVID-19 has the potential to substantially exacerbate socioeconomic and ethnic inequalities in health, unless measures are taken to mitigate these inequalities [46]. Thus, policy response is essential to guarantee that the health system is reactive to the requirements of these vulnerable groups.

The present study has some important limitations. First, collider bias is an important problem and could result in several ways, including differential healthcare requesting, differential examination and differential diagnosis. Despite this, we did not find evidence to suggest that differential medical care seeking, or testing would explain the pattern of results observed in our study. Second, as we noted in a previous study [34], Although the information is from subjects from all over Mexico, patients who were asymptomatic or who were treated at home are not part of our data, so our study represents only those most severe cases of COVID-19, and the results observed cannot be extrapolated to non-severe COVID-19 cases. Moreover, since sentinel units were not randomly designated, our results are not likely to be representative of the entire Mexican population. Furthermore, at the national level, by protocol, tests were only performed on those patients with severe respiratory conditions, and to a lesser extent on subjects with mild respiratory conditions; therefore, our results may underestimate how living in municipalities with higher rates of crowding and higher rates of poverty are associated with the COVID-19 prognosis, as it is highly likely that people living in municipalities with higher rates of crowding and higher rates of poverty have less access to health services. In addition, in relation of the association with non-communicable diseases, the protocol mentioned above may also underestimate the relationships, as those with mild respiratory conditions will have a better prognosis. Finally, our indicators of crowding, poverty, vulnerabilities, and other conditions, share these indicators at the municipality level; thus, it does not reflect the condition of the individual but that of the municipality where people live.

Although the limitations, the present study has some strengths. Comparative studies between crowding conditions and poverty as well as other vulnerabilities associated with the risk of mortality from COVID-19 are scarce; therefore, our findings may help identify these relations. Although it is probable that our results are not representative of the entire population, our study contains nationwide data. Lastly, as previously reported [34], multivariate analysis, due to our large sample size, reduce de possibility of confounding factors.

## Conclusion

This study adds information that emphasize that vulnerable communities have a greater risk of mortality from COVID-19. Our data suggest that patients living in municipalities with higher rates of crowding conditions, higher rates of poverty, and higher rates of other conditions; indigenous population, illiterate, food insecurity, lack of access to social security, lack of access to health services had higher risk of mortality from COVID-19. Also, we found a joint effect of living in municipalities with higher rates of crowding conditions and living with certain comorbidities like obesity, diabetes, and/or hypertension, and the risk of mortality from COVID-19 in Mexican population.

In Mexico, the COVID-19 pandemic is a systemic crisis linked to human development since we have seen that it affects less developed and more vulnerable localities. Policies to reduce vulnerabilities and develop strategies need to consider the following points: 1) adoption of effective evidence-based prevention mechanisms that consider collective risk and the social context; 2) expand and prepare the health system at all levels; 3) guarantee social protection; and 4) develop strategies that promote vaccination of vulnerable populations.

## Supporting information

S1 Fig Flowchart of the Mexican National Epidemiological Surveillance System for viral respiratory disease. (TIF)

S2 Fig Standardized guideline for Epidemiological and Laboratory Surveillance of viral respiratory disease. (TIF)

S1 Table Sociodemographic characteristics and chronic conditions of the study population. (DOCX)

## List of abbreviations

SARS-Cov2 (COVID-19): severe acute respiratory syndrome coronavirus
SISVER: Epidemiological Surveillance System for viral respiratory disease
USMER: sentinel surveillance health units (Spanish acronym for Unidades de Salud Monitoras de Enfermedad Respiratoria)
CONAPO: Mexican Population Council (Spanish acronym for Consejo Nacional de Población)
CONEVAL: Mexican National Council on Evaluation of Social Policy (Spanish acronym for Consejo Nacional de Evaluación de la Política de Desarrollo Social)
CKD: chronic kidney disease
COPD: chronic obstructive pulmonary disease
SD: standard deviations
HR: Hazard ratio
95% CI: 95% confidence intervals
