# Supplementary figures and images for: Association between living in municipalities with high crowding conditions and poverty and mortality from COVID-19 in Mexico
Source: PLoS One. 2022 Feb 22;17(2):e0264137. doi: 10.1371/journal.pone.0264137 (PMC8863291; doi:10.1371/journal.pone.0264137)

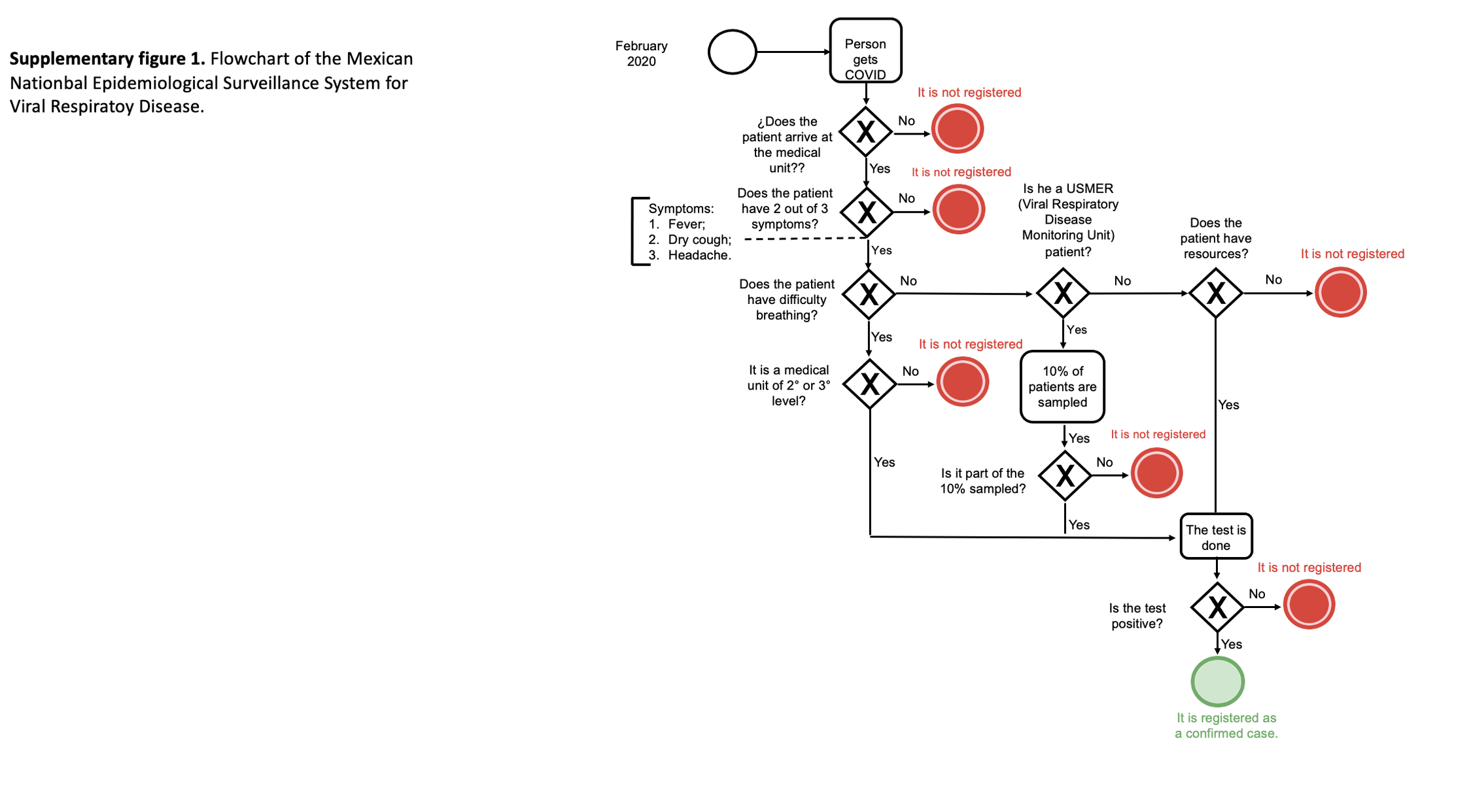

Supplement: S1 Fig — (TIF) [file pone.0264137.s001.tif]

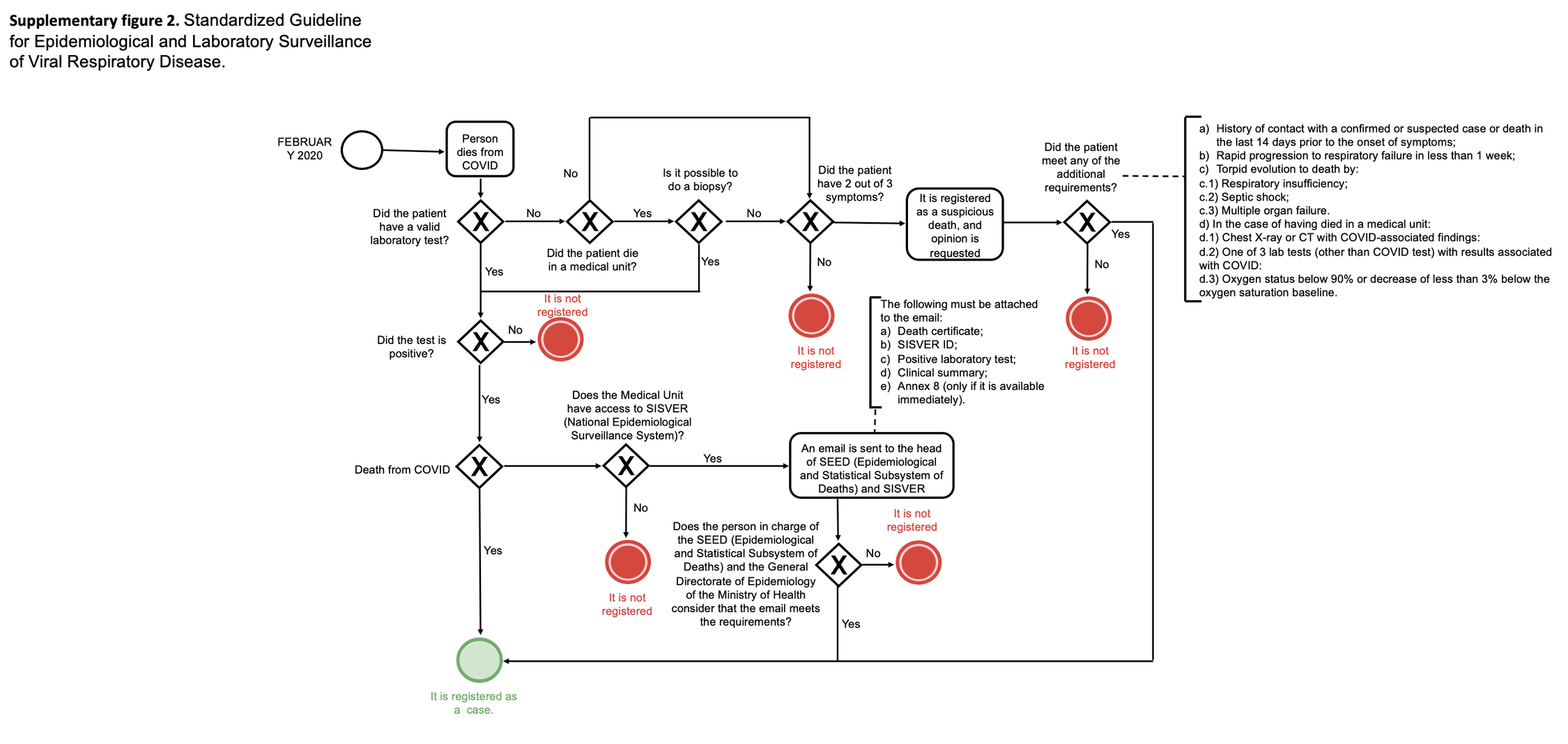

Supplement: S2 Fig — (TIF) [file pone.0264137.s002.tif]
