# Supplementary material for: Association between living in municipalities with high crowding conditions and poverty and mortality from COVID-19 in Mexico
Source: PLoS One. 2022 Feb 22;17(2):e0264137. doi: 10.1371/journal.pone.0264137 (PMC8863291; doi:10.1371/journal.pone.0264137)
Supplement: S1 Table — (DOCX) [file pone.0264137.s003.docx]

**S1 Table 1. Sociodemographic characteristics and chronic conditions of the study population.**

| **Variables** | **Total**  **n=9625528** | **Negative to COVID-19**  **n=6145223** | **Positive to COVID-19**  **n=3480282** |
| --- | --- | --- | --- |
| Age | 43.6 | 40.8 | 46.9 |
| Women, (%) | 47.6 | 46.3 | 50.0 |
| **Individual gaps and vulnerabilities** |  |  |  |
| Indigenous population, (%) | 6.4 | 5.0 | 7.3 |
| Incomplete Elementary school, (%) | 23.0 | 22.7 | 23.6 |
| Illiteracy, (%) | 2.7 | 2.6 | 2.9 |
| Gini index, (%) | 40.0 | 41.0 | 39.0 |
| Lower income welfare line, (%) | 42.8 | 42.6 | 43.3 |
| Lower income minimum welfare line, (%) | 11.8 | 11.7 | 12.1 |
| Multidisciplinary poverty, (%) | 34.2 | 33.9 | 34.7 |
| Extreme multidisciplinary poverty, (%) | 3.6 | 3.5 | 3.9 |
| Educational backwardness, (%) | 12.8 | 12.5 | 13.3 |
| Vulnerability due to income, (%) | 8.7 | 8.6 | 8.8 |
| Crowding conditions, (%) | 8.1 | 7.8 | 8.4 |
| Lack of access to food, (%) | 17.7 | 17.0 | 18.3 |
| Lack of access to social security, (%) | 48.8 | 48.1 | 50.0 |
| Lack of access to basic housing services, (%) | 9.8 | 9.1 | 10.7 |
| Lack of access to health services, (%) | 17.5 | 17.7 | 17.3 |
| **Chronic conditions** |  |  |  |
| Obesity, (%) | 11.3 | 8.9 | 13.7 |
| Diabetes, (%) | 8.8 | 7.2 | 11.6 |
| Hypertension, (%) | 11.9 | 10.2 | 14.9 |
| Cardiovascular disease, (%) | 1.3 | 1.1 | 1.5 |
| Chronic obstructive pulmonary disease, (%) | 0.9 | 0.8 | 1.1 |
| Chronic kidney disease, (%) | 1.2 | 1.0 | 1.5 |
| Immunosuppression, (%) | 0.8 | 0.7 | 1.0 |
| Asthma, (%) | 2.3 | 2.2 | 2.4 |
